# 

**Published:** 1899-11

**Authors:** 


### CONTENTS.

### COMMUNICATIONS.

The Civilized Mouth ........................................................ 493
Detail in Cleft Palate Operations.—Abstract.............................. 501
The Foreign Relations Committee of the National Association of Dental Faculties .................................................. 526

### PROCEEDINGS.

National Dental Association, Niagara Falls, August 1–4, 1899......... 506
Porcelain Enamel Inlays ........................................ 506
The Radical Cure of Cleft Palate.................................. 509
A Resume of the Important Changes that Have Taken Place in Dentistry during the last Thirty-five Years ... 512

### SELECTIONS.

Nerve Tire in the Schools ................................................ 515
An Enviable Principality ................................................ 517
Influence of Alcohol on Muscular Work .................................. 518
Goethe as an Anatomist .................................................. 518
Stomatitis Due to Milk .................................................. 519
Ocean and High Altitude Health Resorts.................................. 519
Pure Drinking Water...................................................... 520
Observations in Anesthesia............................................... 520
Bacteriology............................................................. 521
Cause of Lamp Explosions ................................................ 521
Doctors and Health....................................................... 522
Nitrous Oxid Gas and Oxygen Anesthesia.................................. 522
The Voice and the Circulation............................................ 523
Early Decay of Teeth in Britain ......................................... 523
"Forced" Education ...................................................... 524
Preservation of Cadavers for Anatomic Studies............................ 524
Disinfection of Railway Coaches.......................................... 525
Toothache ............................................................... 525
All Hypodermic Injections may be Rendered Less Painful ............ 525
The Illinois State Board of Health....................................... 526
University for Women in Moscow........................................... 532

### NOTICES.

International Dental Congress, Paris, France, August 8–14, 1900....... 532
Transportation Committee................................................. 533

### EDITORIAL.

The Thirty-fourth Annual meeting of the Ohio State Dental Society 534
Look Out! Under $45,000 Bonds.............................................537

### BIOGRAPHICAL.

Dr. Josiah Ramsey is Dead................................................ 538
Resolutions adopted by the Springfield Dental Association in Memory of the late Dr. Ramsey......................... 540

